# Annotations

**Published:** 1898-04-09

**Authors:** 


### Annotations.

### Alien Immigration.

England has long been an open asylum for all foreigners in distress, and in days when locomotion was less easy than it is at present, and when a considerable proportion of the aliens who landed on our shores had been driven from their homes for political reasons, it was a matter of which England might well be proud that her shores afforded an ever-open way of escape from tyranny and oppression. Things, however, wear a somewhat different aspect when, instead of being men who have left their homes for conscience's sake, to the intermingling of whose blood at various periods of our history the English race owes so much, our immigrants become the sweepings of Europe, and we find our land made a dumping-ground for all that is weak and feeble and disreputable in foreign countries. Under these circumstances we cannot but regard with interest a Bill brought forward by the Earl of Hardwicke, entitled "An Act to Regulate the Immigration of Aliens," however little we may expect it to become law. It provides for inspection of immigrant passengers at certain ports to be specified by Order in Council, and for prohibiting the landing of any alien who, in the opinion of the inspectors, is either an idiot, insane, a pauper, a person likely to become a public charge, or a person suffering from any dangerous, contagious or infectious disease. We do not think that this country will readily give up the position it has so long held as a refuge for the oppressed, but for all that it is difficult to avoid the conclusion that much good would be done by placing some sort of check upon the constant stream of social failures which now seems to be setting so steadily towards our shores.

### Private Lying-in Institutions.

The evolution of the private hospital presents many possibilities, and it is not without interest to consider the future which may lie before the private institution for accouchement. At the present time in England there are, no doubt, many houses whose owners devote themselves, as a sort of specialty, to the reception of ladies during their confinements. They are, however, in most instances mere lodgings even at the best, and at the worst they are something very evil indeed. The private lying-in hospital, under the immediate control and management of the physician accoucheur, is at present non-existent. Yet recent statistics as to the safety with which confinements can be conducted in public hospitals, and many other facts, such as the general domestic disorganisation which is involved when such events occur at home, the doubts and difficulties in engaging a new nurse, the hurry and scurry of fetching the doctor, and the long, weary wait which the medical man has so often to undergo, if he would be sure of doing his best for his patient, all point to the desirability of the lady going to the doctor for her confinement rather than the doctor going to the lady. The statistics of years ago made medical men look upon lying-in hospitals as in every way bad, and only to be put up with as being better than the still worse position of some poor women in their own homes. But things are changed now, and we cannot shut our eyes to the fact that many a lady in good position gets but scant attendance in her confinement at her own home compared with what she might have in an institution. The continued loss of life from puerperal fever shows, indeed, that the security of home is more or less hypothetical; and it certainly is the fact that many a woman who comes up to London, or goes into some large town, for the sake of being near a doctor, is led to be confined among surroundings not equal to those even of her own house. Under these circumstances it seems that there would be many advantages in properly-constituted private lying-in institutions, built after the manner of modern hospitals, and furnished like them with every appliance for the conduct of aseptic midwifery. The days when a medical man's stock-in-trade consisted of a stethoscope and a lancet are far past, and it seems not improbable that the "business side" of the profession may before many years are over assume large proportions, for when men recognise that the private hospital, the private clinic, and the private *maison d'accouchement* not only mean money but fame, and also open a way to the doing of work of a high grade with proper appliances, which after all is the great attraction of a hospital appointment to men of a scientific bent, men of means and of position will be found ready to occupy the field.

### The Plague.

The outbreak of plague at Jeddah is a matter of very considerable interest to Europe. Jeddah, being the port of Mecca, is a half-way house between Europe and farther East, a place of call for pilgrims and a place of exchange for all the infectious diseases from which they may be suffering. Yet Europe has but little say over the doings of this most dangerous plague spot. All attempts to regulate the Mohammedan pilgrimage to this holy city are met by protests against interference with religious sentiments. Yet all the time even the Turks themselves object to the infection of plague being carried in their direction. The gradual onward creeping of the plague should teach us how little we know of the real causes of epidemics. During all this century, until the last few years, plague has been gradually receding from Europe. Places where it was formerly prevalent became free, but in Persia, Kurdistan, and Asia Minor it is doubtful whether it ever passed entirely away, and certainly repeated outbreaks have occurred there during the past fifty years, and spread a certain distance, but never far. Now, however, plague seems again to be taking on a tendency to spread, and although its march is slow, we cannot shut our eyes to the fact that the efforts made to cope with it have had curiously little influence over its progress. Bombay is a melancholy example of our inability to control the disease, and we must not be too sure that where plague has left a district its departure has been due to the measures taken to control it. We know but little about the relation between *pestis minor* and *pestis major*, and, except as regards dirt and overcrowding, we know hardly anything in regard to the influences which increase the virulence of the disease. Now, Southern Europe is by no means free from dirt and overcrowding, and, so far as knowledge goes, European countries have but small reason for assuming that they are safe from plague.

